# A Review of Comparison of Complications of Vaginal Hysterectomy with and without Concomitant Surgery for SUI: A 5 Years' Experience at a Tertiary Care Hospital of Pakistan

**DOI:** 10.1155/2013/540646

**Published:** 2013-12-22

**Authors:** Raheela Mohsin Rizvi, Munnazza Akhtar, Nadeem Faiyaz Zuberi

**Affiliations:** Department of Obstetrics and Gynecology, The Aga Khan University, Stadium Road P.O. Box 3500, Karachi 74800, Pakistan

## Abstract

*Objective*. The study was performed to review the complications of surgery for POP with or without surgery for SUI. This included the need for second procedure two years after the primary surgery. *Study Design*. We conducted a retrospective cross-sectional comparative study at the Aga Khan University, Karachi, Pakistan. International Classification of Diseases, 9th revision, Clinical Modification (ICD-9-CM) was used to identify women who underwent vaginal hysterectomy with anterior/posterior repair alone and those with concomitant tension-free vaginal tape surgery for urodynamic stress incontinence. *Results*. The 28 cases of VH/repair combined with TVT were compared for complications with 430 cases of VH with repair alone. The basic characteristics like age, BMI, and degree of prolapse showed no statistical difference among two groups. The main comorbidities in both groups were hypertension, diabetes, and bronchial asthma. We observed no significant differences in intraoperative and postoperative complications except for cuff abscess, need for medical intervention, and readmission following discharge from hospital, which were higher in cases with vaginal hysterectomy with concomitant TVT. *Conclusions*. Vaginal hysterectomy is an efficient treatment for uterovaginal prolapse with a swift recovery, short length of hospital stay, and rare serious complications. The addition of surgery for USI does not appear to increase the morbidity.

## 1. Introduction

Pelvic organ prolapse (POP) affects millions of women; approximately 200,000 inpatient surgical procedures for prolapse are performed annually in the United States [1, 2]. Eleven to 19 percent of women will undergo surgery for prolapse or incontinence by age 80 to 85 years, and 30 percent of these women will require an additional prolapse repair procedure [3, 4]. The prevalence of POP in rural Pakistan is 12.1% and the prevalence of POP with urinary incontinence (UI) is 12.5% [5].

Pelvic organ prolapse (POP) and stress urinary incontinence (SUI) are common, often coexist, and can greatly impact quality of life [6]. Population-based studies report an 11–19% percent lifetime risk in women undergoing surgery for prolapse or incontinence [5, 7]. Recurrence of symptoms is common, following POP or SUI. Six to twenty-nine percent of women who undergo pelvic floor repair will require additional surgery for recurrent prolapse or stress incontinence [5, 8]. The success observed with the use of surgical mesh in general surgery, combined with the high failure rates for traditional colporrhaphy, has led gynecologic reconstructive surgeons to implement surgical approaches that utilize reconstructive materials [9]. According to medical device manufacturer estimates, approximately 33 percent of POP repair procedures and 80 percent of incontinence procedures utilize reconstructive materials [10]. During 2008, NICE guidelines [11] were published regarding surgical repair of vaginal wall prolapse using mesh. The results of meta-analysis concluded that mesh seemed to improve objective outcome for anterior repair, while subjectively there was no change and, for posterior repair, mesh made no difference. The same group of consultants published systematic review regarding efficacy and safety of mesh or grafts in surgery for anterior and/or posterior vaginal wall prolapse [12]; the meta-analysis showed good outcome in terms of recurrence by the use of nonabsorbable mesh and they concluded that evidence for most outcomes was too sparse to provide meaningful recommendations. Recently, Maher et al. [13] published Cochrane review regarding surgical management of POP and it revealed that the use of mesh or graft inlays at the time of anterior vaginal wall repair reduces the risk of recurrent anterior wall prolapse on examination. The review includes twenty-one trials that compared a variety of surgical procedures for anterior compartment prolapse (cystocele). Ten trials compared native tissue repair with graft (absorbable and permanent mesh, biological grafts) repair for anterior compartment prolapse. Native tissue anterior repair was associated with more recurrent anterior compartment prolapse than when supplemented with a polyglactin (absorbable) mesh inlay (RR 1.39, 95% CI 1.02 to 1.90) or porcine dermis mesh inlay (RR 2.08, 95% CI 1.08 to 4.01).

Pakistan is a developing country with limited resources for the treatment of POP. Even though the Aga Khan University is a well-equipped tertiary level teaching hospital, but the percentage of population who can access treatment in this hospital is very limited. The mesh tools like trocar guided mesh or mesh fixating kits are not readily available; few trocar-guided mesh kits are used for stress urinary incontinence and custom-tailored mesh is used for recurrent or vault prolapse cases.

In view of serious concerns with the use of mesh, we planned a retrospective study to review the complications of simple vaginal hysterectomy (VH) with anterior and posterior repair along with McCall's culdoplasty for prevention of vault prolapse and concomitant surgery for SUI.

## 2. Materials and Methods

We conducted a retrospective cross-sectional comparative study at the Aga Khan University, Karachi, Pakistan. International Classification of Diseases, 9th revision, Clinical Modification (ICD-9-CM) procedure codes 68.59 and 59.79 were used to identify women who underwent vaginal hysterectomy with anterior/posterior repair alone and those with concomitant tension-free vaginal tape surgery for urodynamic stress incontinence. ICD-9-CM procedure codes were used to estimate these procedures, from January 2005 till December 2010. At our hospital, standard definition and classification for the degree of POP and UI is used. We use the Baden-Walker classification for the grades of anterior and posterior vaginal wall and for uterine prolapse; hence, in all cases, preoperative evaluation was made in clinics using the Baden-Walker classification for grades of POP and it was further confirmed under anesthesia. The study was conducted by reviewing medical records of all the women who underwent vaginal hysterectomy for pelvic organ prolapse. We assessed the outcome of VH among two groups, one had simple VH with anterior and posterior repair (*n* = 430), while the other group had similar surgery along with midurethral sling operation for SUI (*n* = 28) and McCall's culdoplasty was routinely performed in all cases for prevention of vault prolapse.

### 2.1. Surgical Complications

The following were noted as intraoperative complications: hemorrhage, urinary bladder, ureter, and rectal injury. We also included postoperative early complications, (within 7 days of surgery) prolong length of stay, unplanned ICU admission, blood transfusion (s), urinary tract infection, urinary retention, pelvic infection, nonpelvic infection, deep venous thrombosis (DVT) and febrile illness. Postoperative delayed complications (between day 8 and 6 weeks completed) included vaginal vault hematoma, cuff abscess, readmission, need for laparoscopy, voiding dysfunction, need for a second procedure for POP, and medical interventions (including extended antibiotic usage, use of low molecular heparin, and extended usage of analgesia). Postoperative delayed complications (between 6 weeks and 2 years completed) included repeat surgery for POP and/or surgery for SUI.

### 2.2. Statistical Analysis

Entry of data and analysis was done using SPSS version 20. Descriptive statistics were computed for all variables of the study. Comparisons between two groups were evaluated with Chi-square or Fisher's exact test for nominal variables and the 2-sample *t*-test. All calculated *P* values were 2 sided, and *P* less than 0.05 was considered statistically significant.

## 3. Results

Results showed that there were 28 cases of VH/repair along with TVT compared to 430 cases of VH with repair. The incidence of complications in our cohort with uterine prolapsed managed by native tissue repair was low.  Table 1 shows patients' characteristics and preoperative evaluation of prolapse. Both groups showed no statistical difference in terms of age and BMI. Vaginal hysterectomy and vaginal hysterectomy with TVT had no difference in preoperative evaluation regarding anterior defect and uterine prolapse; however, posterior defect had statistically significant difference (*P* = 0.046). The medical comorbidity of both groups is summarized in Table 2. The main comorbidity identified is hypertension and it is the same in both groups with *P* = 0.635, showing no statistical difference. Diabetes mellitus and bronchial asthma were other two large comorbidities which were equal in both groups (*P* = 0.116 and *P* = 0.45, resp.). A total of 68 (63 women with VH and repair and 5 women with VH, repair, and TVT) had history of previous surgery. This included surgery for POP like a Manchester repair, anterior and posterior colporrhaphy, surgery for SUI like a Marshall-Marchetti-Krantz, Burch colposuspension, the obstetric procedure like cesarean sections.

The American Society of American Society of Anesthesiologists score was also the same in both groups (*P* = 0.428).  Table 3 shows intraoperative and postoperative complications in both groups. We observed that there was no significant difference in intraoperative and post-operative complications except for cuff abscess, need for medical intervention, and readmission following discharge from hospital, which were higher in cases with vaginal hysterectomy with concomitant TVT (*P* values 0.48, 0.001, and 0.001, resp.).

We demonstrated excellent prolapse recurrence rates with only two women (0.2%) requiring repeat surgery.

## 4. Discussion

This study highlights that the morbidity of vaginal hysterectomy and repair with or without concomitant surgery for SUI is very low. Patients referred to our tertiary care center commonly present with a greater number of clinically important comorbid conditions and greater degree of uterovaginal prolapse. The main findings are the vaginal hysterectomy performed for vaginal prolapse in routine clinical health care setting results in short hospital stay, swift recovery, and a low rate of complications whether performed alone or with concomitant surgical procedure for SUI. Furthermore, there is a high success rate in terms of need for a second procedure and the results are comparable to those of other studies of vaginal hysterectomy, regardless of indication [5, 14–17].

Our study has some limitations. First, it was inherently limited by its retrospective nature. Second, there was a disparity in number of cases in both groups although some of the basic demographic characteristics were statistically different, but these differences were overall clinically similar in both groups. Third, we examined followup data only through the 2 years of postoperative period and complications like vault prolapse usually appear late; however, complications due to surgery should diminish with time.

The strength of the study is that all cases were operated by experienced gynecologists and medical records included clear indication types and grades of prolapse. All the cases with stress urinary incontinence had urodynamic studies to establish the diagnosis of urodynamic stress incontinence (USI). Thus, the data used in the current study was without any greater bias or flaw.

The main comorbidities identified in our study were hypertension (42%), diabetes mellitus (21%), bronchial asthma (7.2%), and anemia (7%). Our study participants had higher frequency of hypertension and diabetes as compared to the prevalence of type 2 diabetes mellitus in Pakistan as 7–11% [18] and hypertension as 21.5% [19]. A study from Nepal showed that 35% of women with POP had a chronic obstructive pulmonary disease (COPD), 16% suffered from hypertension, and 5% had diabetes mellitus [20]. There was a history of previous surgery either abdominal or vaginal (15%) including 5 cases of VH with TVT and 63 cases of VH with repair, but this did not lead to increased morbidity; in an Indian study, a similar number of cases (15.2%) had a prior history of surgery and in one case vaginal hysterectomy route was changed to abdominal hysterectomy [21]. In another study from Sweden, conversion to laparotomy was performed in two cases, one due to complication because of bleeding and the other because of adhesions, narrow vagina, and immobile uterus [22]. In the current study, intraoperative hemorrhage occurred in a total of 19/458 cases; however, there was no need for reoperation and all these cases required only blood transfusion. Swedish study showed the need for reoperation during hospital stay in 1% of patients primarily due to retroperitoneal and intra-abdominal bleeding [22]. Among the delayed complications, cuff abscess occurred in 2 cases of VH with repair and underwent diagnostic laparoscopy. While in one case of VH with repair and TVT, cuff abscess was managed conservatively. The prevalence of infections associated with vaginal hysterectomy for POP during hospital stay has been estimated at 2% for vaginal infections, 1.3% for urinary tract infections (UTI), and 0.1% for combined genitor-urinary infections [22]. Most of our patients received only preoperative prophylactic antibiotics. Febrile illness was reported as an early complication in 12/458 (2.6%) of total cases with increasing frequency in both groups (*P* = 0.006). Urinary tract infection was diagnosed in 9 women and in 3 cases there was unspecified infection; however, this led to prolonged length of stay. The risk of bladder injury during vaginal hysterectomy has been reported as 13.1 per 1000 [23]. There was one bladder perforation in a case of TVT with an anticipated risk due to previous Manchester repair, VH, and Marshall-Marchetti-Krantz operations. Gilmour and Flowerdew reported the estimated rate of bladder injury associated with other gynecologic or urogynecologic surgeries as 11.2 per 1000 surgeries and the rate of ureteral injury as 2.2 per 1000 surgeries [24]. We did not encounter any ureteric injury. The risk of urinary retention and voiding dysfunction is increased with concomitant surgery for SUI. In our study, 9 cases had urinary retention which occurred secondary to urinary tract and pelvic infection, while transient voiding dysfunction with reduced rate of urinary flow occurred in 2 cases of VH-TVT group. In a similar comparative study of VH alone and with TVT, postoperative urinary flow was found lower in the TVT- hysterectomy group: 14 versus 24 mL/s (*P* = 0.02) [25].

Vaginal hysterectomy with repair failed in 2 cases after 12–18 months of surgery and both presented with vault prolapse. Both of these cases initially presented with massive vaginal eversion and refused for vaginal surgery with Mesh. The second procedure required for correction of vault prolapse in these two cases was abdominal sacrocolpopexy with polypropylene mesh and vaginal sacrocolpopexy, respectively. In a retrospective study of 69 patients of vaginal surgery for POP failed in 17 patients during the follow-up at one year and they identified overweight/obesity, high parity, and massive vaginal eversion associated with anatomical and functional failure after POP repair [26].

## 5. Conclusion

Vaginal hysterectomy is an excellent treatment option for uterovaginal prolapse with a swift recovery, shorter length of hospital stay, and rare serious complications. POP and USI can both be safely treated by VH and concomitant surgery for USI. In view of FDA warning for use of mesh, we need to readdress the efficacy of vaginal hysterectomy with concomitant surgery for SUI. A larger prospective randomized trial is required to compare the complication and surgical outcome of VH for POP.

## Figures and Tables

**Table 1:** Characteristics of cases (vaginal hysterectomy with TVT) and controls (vaginal hysterectomy).

Variable	(Vaginal hysterectomy) *N* = 430 (93.9%)	(Vaginal hysterectomy with TVT) N = 28 (6.1%)	*P* value	Total
Age (years)	56.0 ± 11.2	47.8 ± 9.3	0.000	458
Body mass index (BMI)	26.4 ± 4.6	28.4 ± 4.6	0.033	458
Preoperative evaluation of prolapse				
Anterior defect				
Grade 1	139 (32.3 %)	13 (46.4%)	0.168	152 (33%)
Grade 2	124 (28.8%)	4 (6.25%)		128 (28%)
Grade 3	167 (38.8%)	11 (39.2%)		178 (39%)
Posterior defect				
Grade 1	137 (31.8%)	15 (53.5%)	0.046	152 (33%)
Grade 2	121 (28.1%)	7 (25%)		128 (28%)
Grade 3	172 (40%)	6 (21.4%)		178 (39%)
Uterine defect				
Grade 1	137 (31.8%)	18 (64.2%)	0.001	155 (34%)
Grade 2	114 (26.5%)	7 (25%)		121 (26%)
Grade 3	179 (41.6%)	3 (10.7%)		182 (40%)

**Table 2:** Comorbidities of cases (vaginal hysterectomy with TVT) and controls (vaginal hysterectomy).

Variable	Controls (vaginal hysterectomy) n = 430	Cases (vaginal hysterectomy with TVT) n = 28	P value	Total
Cardiovascular				
Hypertension	180 (41.9%)	13 (46.4%)	0.635	193 (42%)
Cardiovascular disease	13 (3%)	0 (0%)	0.351	13 (3%)
Peripheral vascular disease	2 (0.5%)	0 (0%)	0.7	2 (0%)
Myocardial infarction (history)	1 (0.2%)	0 (0%)	0.798	1 (0%)
Respiratory				
Asthma	30 (7%)	3 (10.7%)	0.459	33 (7%)
Obstructive pulmonary disease	1 (0.2%)	0 (0%)	0.798	1 (0%)
Gastrointestinal				
Reflux disease	3 (0.7%)	0 (0%)	0.657	3 (1%)
Bowel disorder	1 (0.2%)	0 (0%)	—	1 (0%)
Liver disorder	1 (0.2%)	0 (0%)	0.798	1 (0%)
Endocrine				
Diabetes mellitus	85 (19.8%)	9 (32.1%)	0.116	94 (21%)
Thyroid disorder	14 (3.3%)	0 (0%)	0.332	14 (3%)
Neurologic cerebrovascular accident	3 (0.7%)	0 (0%)	0.657	3 (1%)
Hematologic anemia (hemoglobin, <11.0 g/dL)	31 (7.2%)	1 (3.6%)	0.464	32 (7%)
History of previous surgery	63 (14.6%)	5 (17.8%)	0.588	68 (15%)
ASA score				
1 or 2	308 (71.6%)	22 (78.6%)	0.428	330 (72%)
3 or 4	122 (28.4%)	6 (21.4%)		128 (28%)

**Table 3:** Complication of cases (vaginal hysterectomy with TVT) and controls (vaginal hysterectomy).

Variable	Controls (vaginal hysterectomy) *n* = 430	Cases (vaginal hysterectomy with TVT) *n* = 28	*P*value	Total (*N*)
Intraoperative complication				
Hemorrhage	18 (4.2%)	1 (3.6%)	0.874	19 (4%)
Urinary bladder injury	1 (0.2%)	0 (0%)	0.798	1 (0%)
Postoperative early complications (within 7 days of surgery)				
Prolong length of stay	14 (3.3%)	1 (3.6%)	0.928	15 (3%)
Unplanned ICU admission	4 (0.9%)	0 (0%)	0.608	4 (1%)
Blood transfusion(s)	20 (4.7%)	0 (0%)	0.243	20 (4%)
Urinary tract infection	7 (1.6%)	2 (7.1%)	0.442	9 (2%)
Urinary retention	9 (2.1%)	0 (0%)	0.439	9 (2%)
Pelvic infection	3 (0.7%)	1 (3.6%)	0.113	4 (1%)
Nonpelvic infection	1 (0.2%)	1 (3.6%)	0.009	2 (0%)
Febrile illness	9 (2.1%)	3 (10.7%)	0.006	12 (3%)
Postoperative delayed complications (between day 8 and 6 weeks)				
Cuff abscess	2 (0.5%)	1 (3.6%)	0.048	3 (1%)
Readmission	4 (0.9%)	2 (7.1%)	0.001	6 (1%)
Need for laparoscopy	2 (0.5%)	0 (0%)	0.718	2 (0%)
Medical interventions	4 (0.9%)	2 (7.1%)	0.001	6 (1%)
(between 6 weeks and 2 years ) need for 2nd procedure for POP	2 (0.2%)	0 (0%)	0.718	2 (0%)
